# To the Editors of the Medical and Physical Journal

**Published:** 1802-09-01

**Authors:** R. Jenkinson

**Affiliations:** Sutton Ashford, Nottingham


### *To the Editors of the Medical and Phyſical Journal.*

GENTLEMEN,

IN looking over an abſtract from Dr. De Carro's "Obſervations on Inoculation for the Cow-pox," in the Appendix to the 37th vol. of the Monthly Review, and from ſome doubts expreſſed in your truly valuable Journal, by Dr. Pearſon, &c. reſpecting the poſſibility of receiving the Vaccine after Variolous infection, I am induced to ſend you the preſent ſtatement as authentic, which, if you think worthy a page in your next Number, you will inſert it, and oblige,

*Sutton Ashford, Nottingham,* Your's, &c.

*August* 10, 1802. R. JENKINSON.

In the beginning of June, 1800, I received two lancets from a medical friend, armed with Vaccine virus, with which, in the courſe of a week, I inoculated three children, (moiſtening the lancet, as I had been in the habit of doing for the variolous, with a little warm water). None of them taking effect, and not having made uſe of the Vaccine virus previous to this time, I felt conſiderably diſappointed, and determined to try its effects another way; accordingly, with one of the ſame lancets, (without moiſtening) I inoculated a perſon who had had the variolous diſeaſe twelve or fourteen years before, in the natural way, which produced a well-formed pock, as I have ever ſince ſeen; and with the matter taken from which, on the 8th and 9th days, the former children were again inoculated, as well as ſeveral others, with the moſt complete ſucceſs, having ſince ſtood the teſt of Inoculation for, and expoſure to, Variolous infection, without producing any effect whatever.

